# Breast cancer prognosis is better in patients who develop subsequent metachronous thyroid cancer

**DOI:** 10.1371/journal.pone.0215948

**Published:** 2019-05-01

**Authors:** Kefeng Lei, Xujun He, Leibo Yu, Chao Ni, Hailong Chen, Dandan Guan, Kewang Sun, Hai Zou

**Affiliations:** 1 Department of General Surgery, Zhejiang Provincial People's Hospital, Hangzhou Medical College, Hangzhou, Zhejiang, China; 2 Department of General Surgery, the 7th Affiliated Hospital of Sun Yat-Sen, University, ShenZhen, Guangdong, China; 3 Key Laboratory of Gastroenterology of Zhejiang Province, Zhejiang Provincial People's Hospital, Hangzhou Medical College, Hangzhou, Zhejiang, China; 4 Department of Pathology, Duke University Medical Center, Durham, NC, United States of America; 5 Mechanical and Electrical Engineering Institute, Jianghan University, Wuhan, Hubei, China; 6 Department of Cardiology, Zhejiang Provincial People's Hospital, Hangzhou Medical College, Hangzhou, Zhejiang, China; King Faisal Specialist Hospital & Research center, SAUDI ARABIA

## Abstract

Breast cancer (BC) and thyroid cancer (TC) are common malignancies among females. However, the connection between TC and BC is not well understood. To explore the relationship between these two cancers and to determine the effect of second metachronous TC on BC survival, we compared BC patients with or without second primary TC using data from the Surveillance, Epidemiology, and End Results (SEER) database. We extracted data from patients with only BC or TC and from BC patients with a second metachronous cancer from 2000–2014. Differences in the clinicopathological and treatment characteristics between BC patients with or without second metachronous TC were analyzed by chi-square tests. Multivariate analyses of BC survival were performed by using Cox regression models. Comparison of disease-specific survival (DSS) curves between these cohorts was performed with the log-rank (Mantel-Cox) test. Survival analyses were also performed using data from 1980–1994. Within this dataset, we found 1,262 BC cases in which a second metachronous TC (BC2TC) developed, accounting for 3.1% of all metachronous cancers following BC from 2000–2014. No significant differences were found in molecular markers. In addition, the mean age at BC diagnosis was younger in the BC2TC group than in the BC group (55.418 y vs 60.273 y). Half of the BC2TC patients developed TC in the first three years following BC diagnosis. Patients with BC2TC showed better DSS than those with BC alone from 2000–2014 (*P*<0.001). However, this superiority was not significant from 1980–1994 (*P* = 0.579) or for TNM stage I BC (*P* = 0.927) and grade I BC (*P* = 0.431) from 2000–2014. In conclusion, the incidence of BC2TC has increased dramatically during the past 15 years. In addition, patients with BC2TC showed better DSS than patients with BC alone, especially in cases from 2000–2014.

## Introduction

Breast cancer (BC) and thyroid cancer (TC) are common malignancies. Although the mortality rate of BC fell between 1989 and 2014, BC remains the second most common cause of cancer-related death among females [1]. In addition, the incidence of TC in the United States has tripled over the past 30 years, with a particularly rapid rise since the 1990s [2].

Many studies have attempted to demonstrate an association between thyroid disease and BC as both diseases principally appear in women. Smyth reviewed the association between thyroid disease and BC and confirmed that hypothyroidism or autoimmune thyroid disease potentially contributes to an increased risk or alters the outcome of BC [3]. In addition, the overall risk of second primary TC or BC is increased in patients with prior BC or TC, respectively [4–6]. However, the interrelationship between TC and BC has not been definitively elucidated [7].

The criteria for diagnosing a second primary tumor or multiple primary tumors have been discussed for many years [8]. Metachronous cancer is considered a secondary cancer diagnosed more than 6 months after the primary cancer diagnosis [9, 10]. In this study, we used data from the Surveillance, Epidemiology, and End Results (SEER) database, which followed the SEER definition of multiple primary tumors [11].

In 1973, Moossa considered that patients with BC and a history of thyroid disease had a lower average survival rate at both 5 and 10 years than those without any evidence of thyroid disease [12]. In addition, it seems logical to consider that adding TC to primary BC would result in a worse prognosis than that of BC alone, but is this true? Here, we used data from the SEER database to analyze the incidence rate of second malignancies following BC and the differences in the clinical, pathological and treatment characteristics and disease-specific survival (DSS) of BC with or without second primary TC. Our results show that the incidence of TC as a second malignancy following BC has increased dramatically during the past 15 years and that BC patients with a second TC have better DSS than patients with BC only, especially in the past 15 years.

## Materials and methods

We used data from the SEER Cancer Registry database, which collects data on every case of cancer reported from 19 U.S. geographical areas. These areas cover approximately 34% of the U.S. population and are representative of the demographics of the entire U.S. population (https://seer.cancer.gov/about/factsheets/SEER_Overview.pdf). We used the following database: Incidence—SEER 18 Regs Custom Data (with additional treatment fields), submitted November 2016 (1973–2014 varying)—Linked to County Attributes—Total U.S., 1969–2015 Counties, National Cancer Institute, DCCPS, Surveillance Research Program, released April 2017. The majority of patients in this study were diagnosed between 1 January 2000 and 31 December 2014. We extracted three cohorts of patients with definite survival months from this database. For cohort one, the total number of in situ/malignant tumors for each patient was 2, with BC as the first malignancy and a second metachronous cancer. In the other cohorts, each patient had only BC or TC. There were 40,520 patients in cohort one, 629,976 patients with only BC and 120,339 patients with only TC. We also extracted cases from 1 January 1980 to 31 December 1994 following the former formula with the second primary TC diagnosed before 1995. All data from SEER database were fully anonymized. The patient records used in our study are provided as supporting databases. The S1 database provides records of the BC cohort from 2000–2014. The S2 database provides records of other cohorts from 2000–2014. The S3 database includes data from all cohorts from 1980–1994.

The following parameters were evaluated for BC: age, race, TNM stage, tumor grade, estrogen receptor (ER) expression, progesterone receptor (PR) expression, Her-2 expression, histopathological subtype, radiation therapy and chemotherapy history. There were 36,825 cases without definite TNM data for the BC only (BC) group and 60 cases without definite TNM data for the BC followed by TC (BC2TC) group. There were 56,532 cases without definite grade data for the BC group and 90 cases without definite grade data for the BC2TC group. There were some cases lacking ER expression data (58,409 for BC; 131 for BC2TC) or PR expression data (66,077 for BC; 150 for BC2TC). There were 214,258 cases with Her-2 expression data for the BC group and 237 cases for the BC2TC group. Radiation therapy included beam radiation, combinations of beam radiation with implants or isotopes, unspecified radiation methods or sources, radioactive implants, and radioisotopes.

We used the Surveillance Research Program, National Cancer Institute SEER*Stat software (www.seer.cancer.gov/seerstat) version 8.3.5 to obtain the incidence rates of patients with TC only or generate lists of cases with the desired data. Data analysis was performed using GraphPad Prism 7 (GraphPad Software, Inc., CA, USA). The differences in clinical characteristics were analyzed by chi-square test. Multivariate analyses of BC survival were performed by using Cox regression models. Comparison of DSS curves was performed using the log-rank (Mantel-Cox) test. *P*<0.05 was considered significant.

## Results

### TC is one of the top ten metachronous cancers following BC

The results of the incidence ratio analysis of a second metachronous cancer following BC are shown in Table 1. The most common metachronous cancer of BC was second primary BC. TC developed in 1,262 cases, a rate of 3.1%. During the same period, the incidence of TC alone was 9.5 per 100,000, age-adjusted to the 2000 US Standard Population. However, the rate of a second TC after BC was 145.7 per 100,000 (1,262/865,886). The total number of BC cases was 865,886 from the database.

**Table 1:** The top ten types of second metachronous cancer (SMC) in breast cancer patients.

SMC type	No.	Percentage (%)
Breast	14,321	35.3
Lung and Bronchus	5,207	12.9
Colon/Rectum	3,683	9.1
Corpus Uteri	2,448	6.0
Skin	1,476	3.6
Pancreas	1,300	3.2
NHL	1,263	3.1
Thyroid	1,262	3.1
Leukemia	1,246	3.1
Ovary	1,153	2.9

### Clinicopathological characteristics of the BC and BC2TC groups

**Fig 1:**
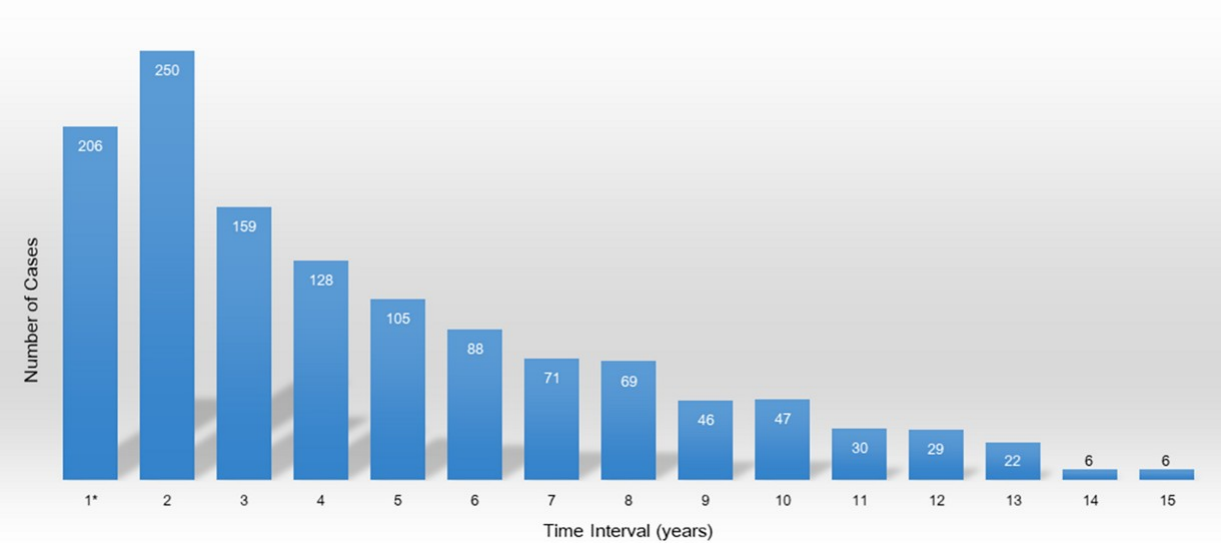
Time interval between breast cancer and thyroid cancer 1*: Only the second half of the first year.

The clinicopathological characteristics of BC were compared between the BC and BC2TC groups. There were 629,976 cases in the BC group and 1,262 cases in the BC2TC group. Between these groups, significant differences were found in race, TNM stage, tumor grade, histological classification, radiation therapy and chemotherapy history but not in molecular markers including ER, PR and Her-2 expression (Table 2). In addition, the mean age at BC diagnosis was younger in the BC2TC group than in the BC group (55.418 y vs 60.273 y, respectively). The numbers of patients who developed TC during the second half of the first year, the second and the third year after BC diagnosis were 206, 250 and 159, respectively (Fig 1).

**Table 2:** Clinical characteristics of patients with only BC (BC group) and those with BC followed by TC (BC2TC group).

Variable	BC (n = 629,976)	BC2TC (n = 1,262)	chi-square P-value
Race			*P*< 0.001
White	506,105 (80.3%)	1,010 (80.0%)	
Black	67,537 (10.7%)	102 (8.1%)	
Other	51,708 (8.2%)	145 (11.5%)	
Unknown	4,626 (0.7%)	5 (0.4%)	
TNM Stage			*P*< 0.001
IV	32,393 (5.1%)	18 (1.4%)	
III	77,388 (12.3%)	172 (13.6%)	
II	209,060 (33.2%)	451 (35.7%)	
I	274,310 (43.5%)	561 (44.5%)	
Grade			*p* = 0.044
IV	6,765 (1.1%)	23 (1.8%)	
III	203,557 (32.3%)	428 (33.9%)	
II	241,996 (38.4%)	494 (39.1%)	
I	121,126 (19.2%)	227 (18.0%)	
ER-positive			*p* = 0.234
No	115,782 (18.4%)	213 (16.9%)	
Yes	455,785 (72.3%)	918 (72.7%)	
PR-positive			*p* = 0.539
No	177,243 (28.1%)	340 (26.9%)	
Yes	386,656 (61.4%)	772 (61.2%)	
Her-2-positive			*p* = 0.062
No	180,368 (28.6%)	189 (15.0%)	
Yes	33,890 (5.4%)	48 (3.8%)	
Breast histology			*p* = 0.036
Infiltrating ductal carcinoma	452,423 (71.8%)	898 (71.2%)	
Infiltrating lobular carcinoma	50,069 (7.9%)	79 (6.3%)	
Mixed invasive	60,781 (9.6%)	146 (11.6%)	
Inflammatory	3,953 (0.6%)	6 (0.5%)	
Other	62,750 (10.0%)	133 (10.5%)	
Radiation therapy			*p* = 0.033
Yes	307,043 (48.7%)	653 (51.7%)	
No/Unknown	322,933 (51.3%)	609 (48.3%)	
Chemotherapy			*P*< 0.001
Yes	260,797 (41.4%)	637 (50.5%)	
No/Unknown	369,180 (58.6%)	625 (49.5%)	

### Survival analysis

Multivariate analyses of BC survival were performed by using Cox regression models for SMC patients first. Second primary TC significantly influenced both OS and DSS of BC (with a second metachronous TC vs. without a second metachronous TC: hazard ratio [HR] 0.248, 95% confidence interval [CI] 0.209 to 0.294, *P*<0.001 for OS; [HR] 0.227, 95% [CI] 0.181 to 0.284, *P*<0.001 for DSS) (S1 Table).

**Fig 2:**
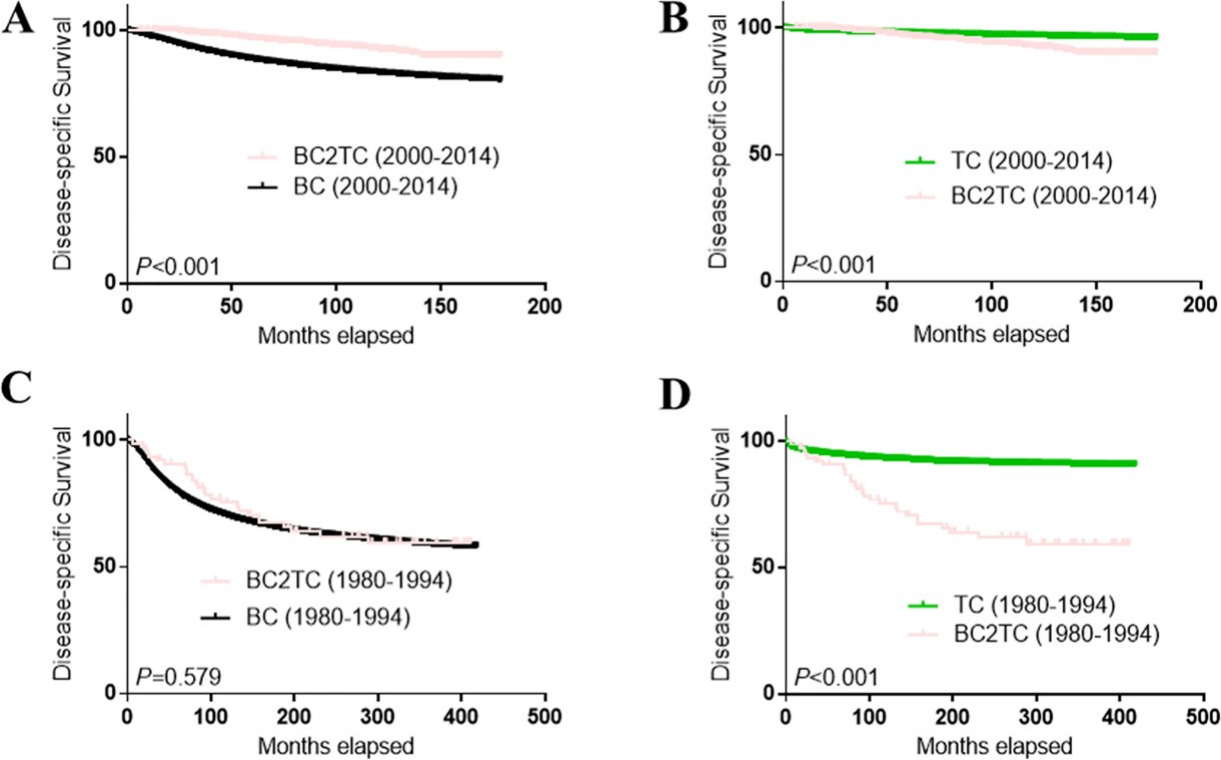
Comparison of DSS among the BC2TC, BC and TC groups. BC vs BC2TC from 2000 to 2014 (A); TC vs BC2TC from 2000 to 2014 (B); BC vs BC2TC from 1980 to 1994 (C); TC vs BC2TC from 1980 to 1994 (D).

**Fig 3:**
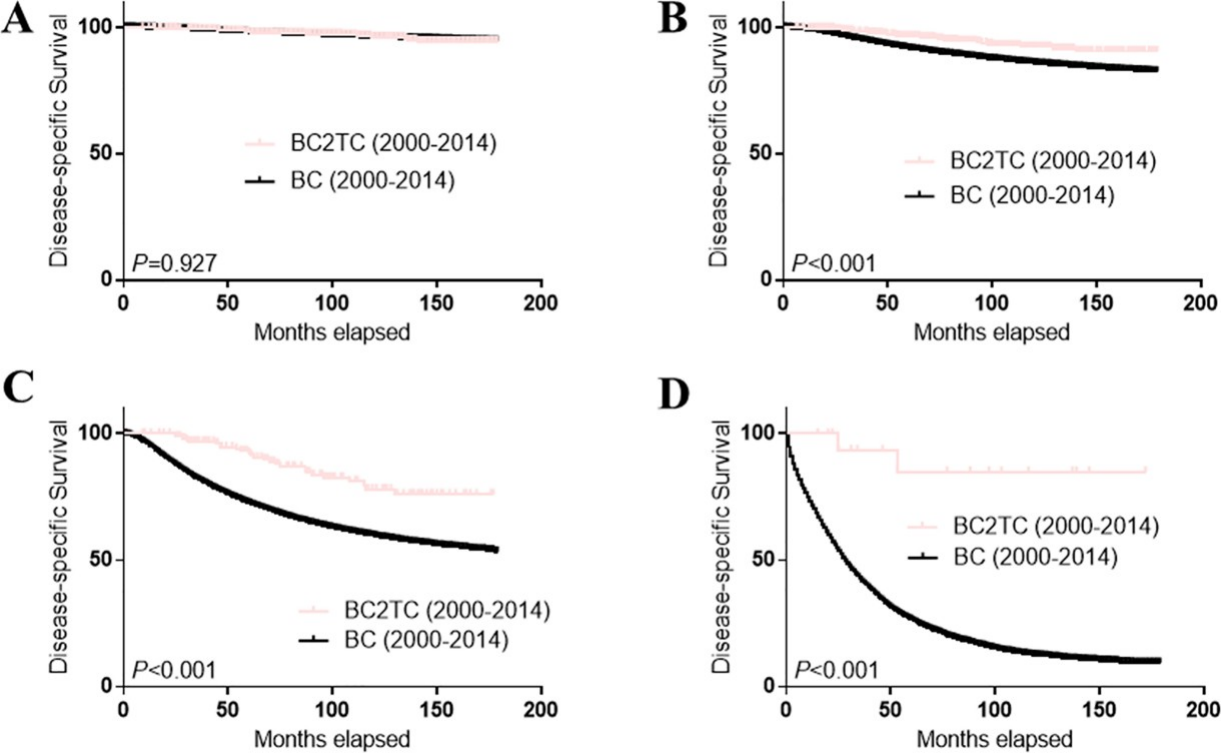
Comparison of DSS among different TNM stages in the BC2TC and BC groups. BC vs BC2TC for stage I (A); BC vs BC2TC for stage II (B); BC vs BC2TC for stage III (C); BC vs BC2TC for stage IV (D).

**Fig 4:**
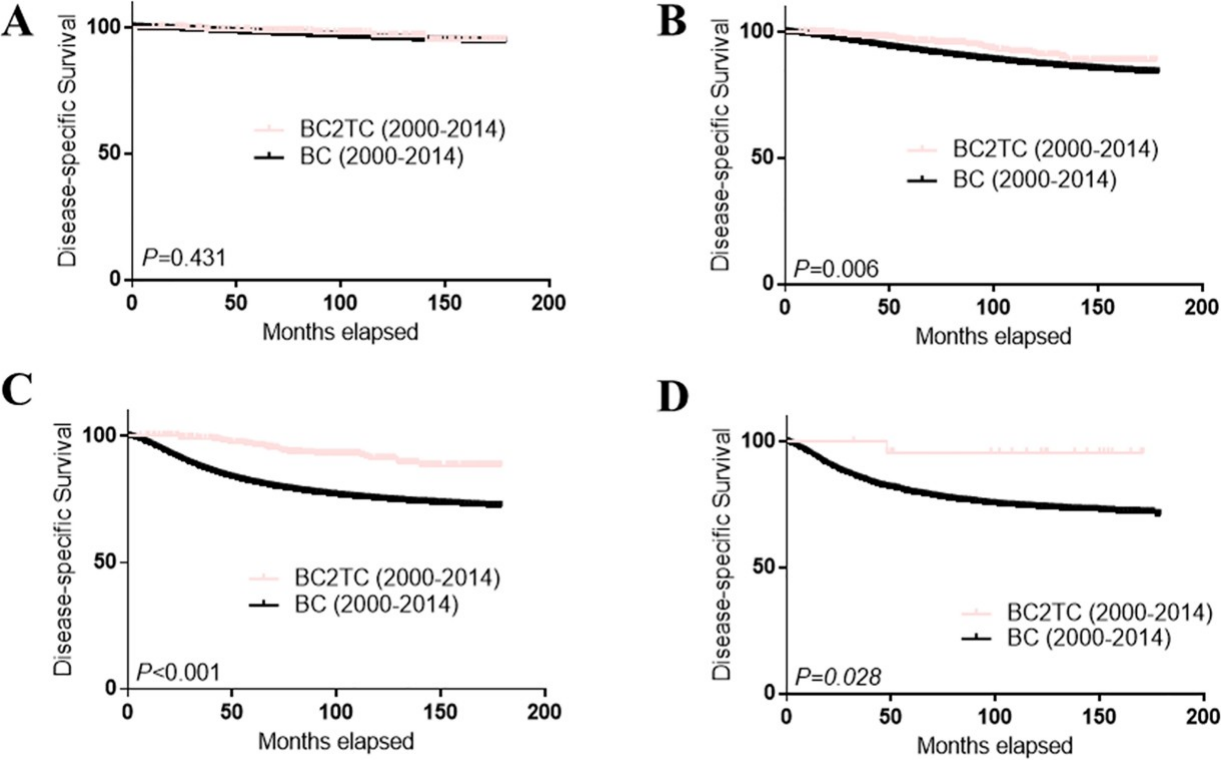
Comparison of DSS among different grades in the BC2TC and BC groups. BC vs BC2TC for grade I (A); BC vs BC2TC for grade II (B); BC vs BC2TC for grade III (C); BC vs BC2TC for grade IV (D).

The survival curves of the BC2TC, BC and TC groups (TC only patients) are shown in Fig 2. DSS in the BC2TC group was much better than that in the BC group (A) but worse than that in the TC group (B) from 2000–2014. However, the survival advantage in the BC2TC group over the BC group was not statistically significant from 1980–1994 (C) (*P* = 0.579) (Fig 2). When we stratified the cases from 2000–2014 by TNM stage (Fig 3) and tumor grade (Fig 4), we observed a significant survival advantage for all groups except stage I (*P* = 0.927, Fig 3A) and grade I (*P* = 0.431, Fig 4A). In addition, the improved survival was obvious in all of the cases regardless of radiation therapy (S1 Fig) or chemotherapy (S2 Fig) history.

## Discussion

Is it true that adding TC to primary BC will lead to a worse prognosis than that of BC alone? Our results indicated a better DSS in the BC2TC group than in the BC group, especially for cases between 1 January 2000, and 31 December 2014. In addition, the incidence of TC as a second malignancy following BC has dramatically increased in the last 15 years.

To the best of our knowledge, this is the first paper to address the effect of second TC on BC survival.

Many studies have focused on the possible relationship between BC and thyroid disease, but the results have been conflicting. Hyperthyroidism was found to be associated with an increased risk of BC compared with the general population, and hypothyroidism was associated with a slightly decreased risk of BC compared with the general population. Thus, it appears that thyroid function levels may be associated with BC risk [13, 14]. However, another study found no statistically significant association between hypothyroidism or hyperthyroidism and BC [15]. In addition, using samples collected from participants at one time point after an overnight fast, Chan et al. found no associations of thyroid-stimulating hormone (TSH), free thyroxine (FT4), or anti-thyroid peroxidase (TPO) with BC [16]. Thyroid hormone substitution treatment did not reduce the incidence of BC [17]. Regarding the effect of thyroid disease on BC survival, in the largest and longest observational study to date, no evidence was found for a prognostic role of anti-TPO antibodies and/or thyroid function in moderate-to-high-risk early BC [18]. However, another study showed a positive association between FT4 levels and improved BC survival [19], whereas data over 10 years from one institute showed that patients with co-existing BC and TC generally exhibited worse survival than those with only BC or TC [20]. What is the actual effect of TC as a second cancer following BC? Our data from the SEER database revealed better DSS for patients with BC2TC than for those with BC alone between 2000 and 2014. In addition, while DSS may be more appropriate in terms of prediction, it does not include all causes [21]. We consider our results to be robust and more representative of the population. However, this survival advantage was not significant in data from 1980 to 1994. To explore reasons for this difference, we retrieved data regarding changes in TC therapy and found that TSH suppression using supraphysiologic doses of levothyroxine (LT4) to suppress the level of TSH without hyper- or hypothyroidism has become increasingly prevalent as a treatment for TC in the past 15 years.

In 1994, Mazzaferri found that thyroid hormone therapy confers a distinct outcome advantage in TC; this was a very important long-term study with a major influence on TC treatment [22]. Then, from 1996 to 2009, TSH suppression strategies became increasingly recommended in the American Thyroid Association (ATA) guidelines for TC therapy [23, 24].

However, whether TSH suppression using LT4 is the reason for the better survival for BC in the BC2TC group remains to be determined. As early as 1896, Beatson used thyroid extract to treat BC and explore the relationship between BC and thyroid dysfunction, and determined that they were correlated [25]. Loeser reported the use of thyroid hormone to treat inoperable BC or BC after radical surgery and found that massive thyroid hormone dosages decreased the rate of BC growth [26]. Now that evidence did not suggest that thyroid hormone causes BC [27]. We shift our sights to TSH. TSH is a hormone of the hypothalamus-pituitary-thyroid axis, and the hypothalamus-pituitary-thyroid and hypothalamus-pituitary-gonadal axes are linked [28]. Endogenous estrogens and progestins, which are regulated by the hypothalamic-pituitary-gonadal axis, have been proved to be carcinogenic and increase the risk of breast cancer [29]. Based on these observations, we inferred that TSH suppression therapy might lead to improved survival for BC due to the interaction between the hypothalamus-pituitary-thyroid axis and hypothalamus-pituitary-gonadal axis.

BC is the most common second primary cancer following BC [30], in agreement with our results. In addition, there is a significantly increased risk of TC after diagnosis with primary BC [31, 32]. Compared with Raymond’s results for data before 2000 [33], our data showed that the incidence of TC has increased more than 4-fold. This is consistent with a true increase in the occurrence of TC in the United States [34], and might also reflect an influence of access to care also [35].

Because it was based on data from the SEER database, our study was retrospective. We found that patients in the BC2TC group had better DSS, but we can only speculate that TSH suppression therapy is the reason for the better survival for BC in these patients. In addition, stage I and grade I BC did not show any survival advantages for subsequent TC; thus, studies to identify the BC patients who might in fact achieve improved survival for second metachronous TC should be performed. The results of this study should be verified in a prospective population-based study, and the underlying mechanisms should be investigated.

In conclusion, despite these limitations, our results showed that the incidence of TC as a second malignancy following BC has dramatically increased during the past 15 years. Notably, our study found better DSS in the BC2TC group than the BC group, especially in cases diagnosed between 1 January 2000, and 31 December 2014. We speculate that TSH suppression therapy is the underlying reason for better DSS in the BC2TC group. TSH suppression therapy may be another potential regimen for BC.

## Supporting information

S1 Table Multivariate analyses of OS and DSS in BC patients with SMC. (XLSX)

S1 Fig Comparison of DSS between BC2TC and BC groups with or without radiation therapy. BC vs BC2TC with radiation therapy (A); BC vs BC2TC without radiation therapy (B).
(TIF)

S2 Fig Comparison of DSS between BC2TC and BC groups with or without chemotherapy. BC vs BC2TC with chemotherapy (A); BC vs BC2TC without chemotherapy (B).
(TIF)

S1 Dataset Data from patient records from the SEER database (BC 2000–2014). (RAR)

S2 Dataset Data from patient records from the SEER database (other cohorts 2000–2014). (RAR)

S3 Dataset Data from patient records from the SEER database (cohorts 1980–1994). (RAR)
