# Supplementary figures and images for: Breast cancer prognosis is better in patients who develop subsequent metachronous thyroid cancer
Source: PLoS One. 2019 May 1;14(5):e0215948. doi: 10.1371/journal.pone.0215948 (PMC6493754; doi:10.1371/journal.pone.0215948)

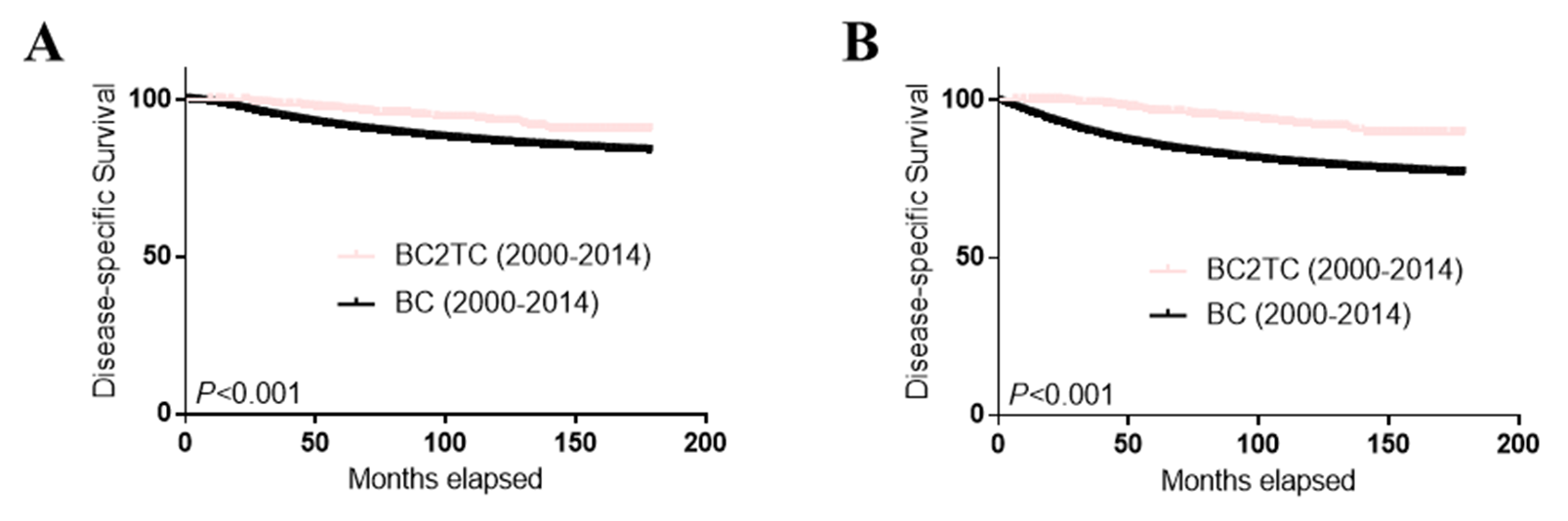

Supplement: S1 Fig — BC vs BC2TC with radiation therapy (A); BC vs BC2TC without radiation therapy (B). (TIF) [file pone.0215948.s002.tif]

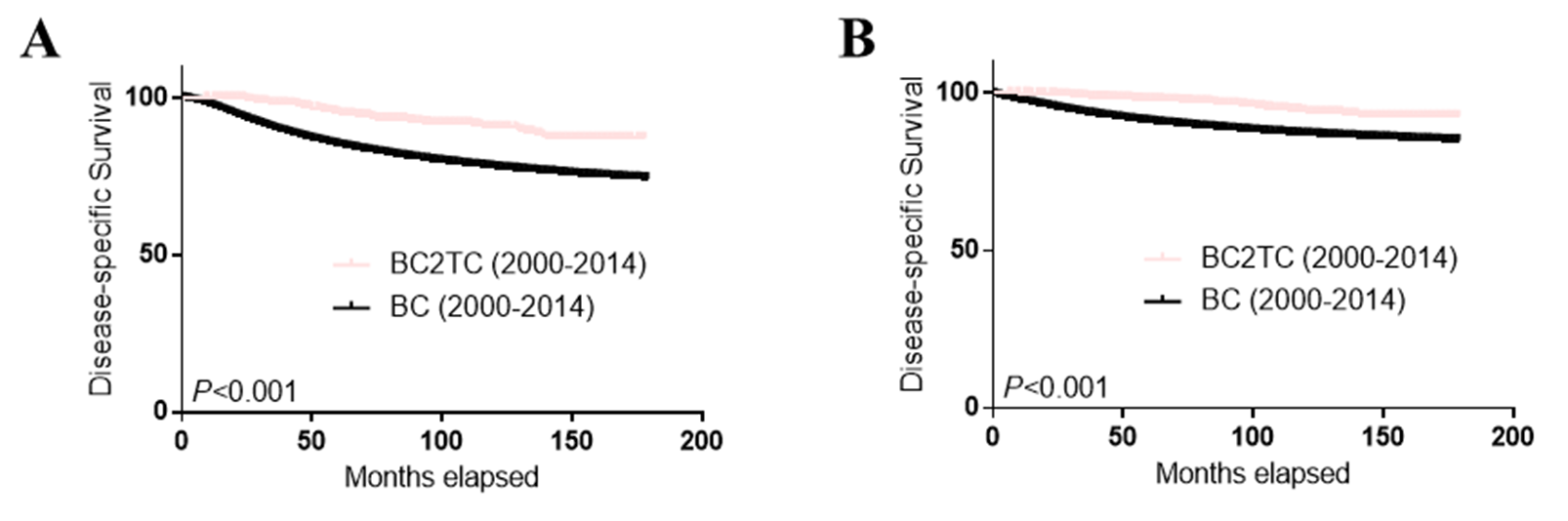

Supplement: S2 Fig — BC vs BC2TC with chemotherapy (A); BC vs BC2TC without chemotherapy (B). (TIF) [file pone.0215948.s003.tif]
